# Inner ear high signal on non‑contrast 3D FLAIR imaging in patients with cerebrospinal fluid leaks: Association with dural/subdural changes and with audiovestibular symptoms

**DOI:** 10.1016/j.ejro.2026.100758

**Published:** 2026-05-04

**Authors:** Iichiro Osawa, Keita Nagawa, Takashi Mitsufuji, Hirokazu Shimizu, Yuya Yamamoto, Shinji Kakemoto, Genko Oyama, Nobuo Araki, Kaiji Inoue, Eito Kozawa

**Affiliations:** a Department of Radiology, Saitama Medical University Hospital, 38 Morohongo, Moroyama-machi, Iruma-gun, Saitama 350-0495, Japan; b Department of Neurology, Saitama Medical University Hospital, 38 Morohongo, Moroyama-machi, Iruma-gun, Saitama 350-0495, Japan

**Keywords:** cerebrospinal fluid leak, 3D FLAIR, hearing loss, tinnitus, diffuse pachymeningeal enhancement, subdural effusion/hematoma

## Abstract

**Background:**

Patients with spinal cerebrospinal fluid (CSF) leaks frequently experience audiovestibular symptoms. While gadolinium-enhanced MRI has been used to detect inner ear abnormalities, no reports have described findings on non-contrast MRI. We aimed to evaluate inner ear high signals on non-contrast three-dimensional (3D) fluid attenuated inversion recovery (FLAIR) imaging and their associations with dural/subdural findings and with audiovestibular symptoms.

**Methods:**

Twenty-one patients (42 ears) diagnosed with CSF leaks were retrospectively recruited. We evaluated inner ear signal intensity on non‑contrast 3D FLAIR imaging in the cochlea, vestibule, and semicircular canals (SCCs) bilaterally, and dural/subdural abnormalities (diffuse dural/subdural hyperintensity and diffuse pachymeningeal enhancement) on head MRI. Associations between inner ear signal and dural/subdural lesions, cochlear signal and auditory symptoms (hearing loss, tinnitus and ear fullness), and vestibular/SCC signal and vertigo were assessed using Fisher’s exact test at the ear level and/or patient level.

**Results:**

Inner ear FLAIR hyperintensity was associated with dural/subdural lesions (*p* = 0.008). Although hearing loss and tinnitus correlated with cochlear hyperintensity in exploratory ear-level analyses (*p* = 0.009 and 0.003, respectively), only tinnitus remained significantly associated at the patient level. Inner ear hyperintensity showed no association with ear fullness or vertigo.

**Conclusions:**

Inner ear hyperintensity on non-contrast 3D FLAIR imaging is observed in CSF leak patients and associates with dural/subdural lesions. Primary patient-level analysis reveals a correlation with tinnitus, while ear-level findings regarding hearing loss remain exploratory. These results may support the utility of non-contrast 3D FLAIR imaging for evaluating inner ear pathology in CSF leak patients.

## 1 Introduction

Spinal cerebrospinal fluid (CSF) leak is associated with a spectrum of etiologies, including spontaneous intracranial hypotension (SIH) [1], traumatic injury, and iatrogenic causes such as lumbar puncture, epidural anesthesia, and spinal surgery [2]. CSF leaks lead to decreased CSF volume and pressure, resulting in various imaging features such as brain sagging and venous dilatation within the skull; subdural effusion/hematoma and diffuse pachymeningeal enhancement can also occur [3]. CSF leaks produce a variety of symptoms, including orthostatic headache and nausea [4]. Importantly, for otologic practice, audiovestibular symptoms (e.g. hearing loss, tinnitus, ear fullness and vertigo) are complicated manifestations caused by CSF leaks [5].

Several mechanisms have been proposed for ear symptoms in patients with CSF leaks, including endolymphatic hydrops [6], traction of the cochleovestibular nerve [7] and dural thickening in the internal auditory canal [8]. Endolymphatic hydrops has been visualized in patients with CSF leaks using delayed contrast-enhanced three-dimensional (3D) fluid attenuated inversion recovery (FLAIR) imaging [9], [10], [11]. Although this imaging method facilitates the detection of endolymphatic hydrops, it yields no data regarding the native inner ear lymphatic environment prior to gadolinium administration.

Non-contrast 3D FLAIR imaging can identify inner ear abnormalities including hyperintensity within lymphatic fluid in patients with ear diseases such as vestibular schwannoma [12], sudden sensorineural hearing loss [13], and Ménière's disease [14]. In particular, heavily T2‑weighted 3D FLAIR (HT2-FLAIR) sequence, a variant of 3D FLAIR sequence, is more sensitive to subtle T1 shortening due to factors such as protein and hemorrhage than conventional 3D FLAIR sequence [12]. For example, HT2-FLAIR imaging allows for the detection of high signal within lymph fluid in patients with vestibular schwannoma, presumably caused by increased protein concentrations within the perilymph [12]. Therefore, inner ear lymphatic hyperintensity may be observed on non-contrast 3D FLAIR imaging in patients exhibiting ear symptoms associated with CSF leaks. Also, this imaging feature may be related to intracranial imaging findings associated with CSF leaks (i.e. subdural effusion/hematoma and pachymeningeal enhancement). However, to the best of our knowledge, no prior reports have described abnormal signal within lymph fluid on non-contrast MRI in these patients.

We noticed high signal in inner ear structures on non-contrast HT2-FLAIR imaging in our CSF leak cohort and hypothesized that such imaging abnormalities would be associated with dural/subdural imaging findings and with audiovestibular symptoms. Therefore, in our study, we aimed to: (1) describe the distribution of inner ear 3D FLAIR hyperintensity in patients with CSF leaks; (2) evaluate association between inner ear hyperintensity and dural/subdural imaging findings; and (3) examine relationships between inner ear hyperintensity and audiovestibular symptoms.

## 2 Materials and methods

### 2.1 Participants

Our study was approved by the Research Ethics Committee of our institution. All experiments were performed in accordance with the relevant guidelines and regulations. We obtained written informed consent for the procedures and opt-out consent for the use of retrospective clinical data from all patients and from parents or legal guardian of patient less than 18 years. We reviewed a consecutive series of 59 patients diagnosed with CSF leaks from November 2018 to November 2023. The etiologies of CSF leaks consisted of SIH (n = 51), lumbar puncture (n = 4), spinal procedure (n = 3) and trauma (n = 1). The diagnosis of SIH, as well as CSF leaks after lumbar puncture, spinal procedure or trauma, was established in accordance with the criteria proposed by the Headache Classification Committee of the International Headache Society, 3rd edition (ICHD-3) [15]. The inclusion criteria were as follows: (1) age ≥ 15 years, (2) diagnosis of CSF leaks according to the criteria proposed by the ICHD‑3, and (3) availability of head and inner ear MRI including dedicated inner ear non‑contrast 3D FLAIR imaging at the time of diagnosis. A total of 23 participants fulfilled the inclusion criteria. The exclusion criteria were as follows: prior otologic disease (n = 1), prior intracranial disease (n = 1), and severe artifacts precluding assessment (n = 0). Finally, data from 21 patients (42 ears) were analyzed.

### 2.2 Clinical data collection

Clinical data were reviewed for presence or absence of: hearing loss, tinnitus, ear fullness, vertigo, and orthostatic headache at the time of MRI. Hearing loss, tinnitus, and ear fullness were evaluated separately for both ears.

### 2.3 MRI acquisition and image analysis

All patients underwent a 3 Tesla MR examination (MAGNETOM Skyra, Siemens, Erlangen, Germany) with a 32-channel head coil (Siemens, Erlangen, Germany) using dedicated inner ear protocol and routine head protocol. The inner ear protocol included axial HT2-FLAIR imaging and axial heavily T2-weighted MR cisternography (MRC), according to a protocol for the evaluation of endolymphatic hydrops [12]: the latter was utilized for anatomical reference of the total lymph fluid. HT2-FLAIR imaging was performed with the following parameters: repetition time (TR) 9000 ms, echo time (TE) 542 ms, inversion time (TI) 2250 ms, frequency-selective fat suppression prepulse, variable flip-angle echo train with an average flip angle of 120° followed by a 90° restore pulse, echo train length 519, matrix size 384 × 384, field of view 166 × 196 mm, axial slices 104, 0.5 × 0.5 mm in-plane resolution at 1.0 mm slice thickness, bandwidth 434 Hz/pixel, an acceleration factor 2 using the generalized autocalibrating partially parallel acquisitions (GRAPPA) parallel imaging technique, number of excitations 2, acquisition time 7 min 17 s. MRC was acquired with the following parameters: TR 4400 ms, TE 542 ms, frequency-selective fat suppression prepulse, variable flip-angle echo train with an average flip angle of 120° followed by a 90° restore pulse, echo train length 519, matrix size 384 × 384, field of view 166 × 196 mm, axial slices 104, 0.5 × 0.5 mm in-plane resolution at 1.0 mm slice thickness, bandwidth 434 Hz/pixel, an acceleration factor 2 using the GRAPPA parallel imaging technique, number of excitations 1.8, acquisition time 3 min 15 s. Among the 21 patients, 15 patients underwent 4-hour delayed post-contrast imaging for the evaluation of endolymphatic hydrops after the non-contrast inner ear protocol, including HT2-FLAIR imaging, MRC, and heavily T2-weighted 3D inversion recovery (HT2-IR) imaging (with TI shortened to 2050 ms, otherwise identical to HT2-FLAIR sequence). 4-hour delayed post-contrast images were obtained 4 h after an intravenous injection of gadolinium-HP-DO3A (Gadoteridol) at a single dose of 0.2 mL/kg (0.1 mmol/kg). By subtracting HT2-IR images from HT2-FLAIR images, HYDROPS (hybrid of reversed image of positive endolymph signal and native image of positive perilymph signal) images were generated, which became possible to recognize endolymphatic space more easily [12]. Among these 15 patients, endolymphatic hydrops was assessed in 13 patients with evaluable images. The head MRI protocol comprised non-contrast FLAIR (TR 5000–12000 ms, TE 94–398 ms, TI 1800–2700 ms, and slice thickness 1.0–5.0 mm) and/or post-contrast T1-weighted (TR 480–587 ms, TE 2.1–10 ms, and slice thickness 5.0 mm) sequences, obtained in axial and/or coronal planes. 3, 11, and 7 patients underwent non-contrast FLAIR imaging, post-contrast T1-weighted imaging, and both types of imaging, respectively. Therefore, dural/subdural lesions were primarily evaluated using post-contrast T1-weighted imaging.

**Fig. 1:**
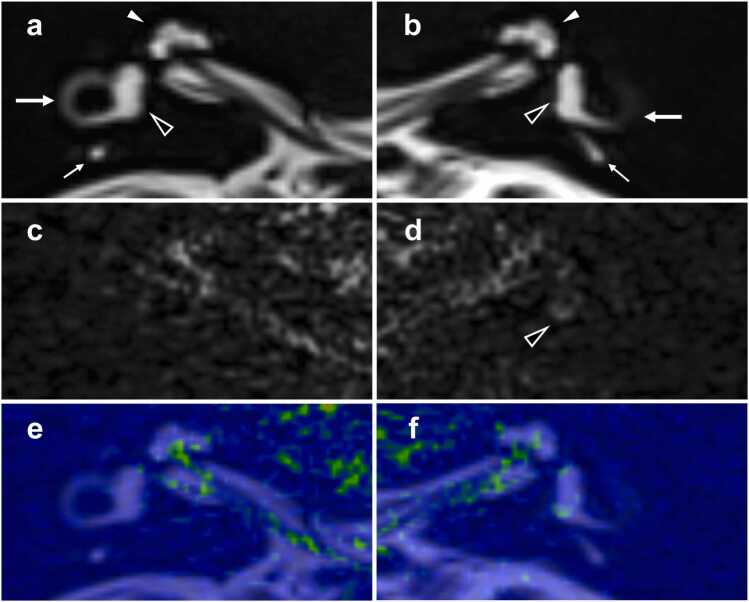
**32-year-old male with ipsilateral inner ear high signal on non-contrast 3D FLAIR imaging. a-b** MRC: Hyperintensity is observed within lymph fluid located in the cochlea (white arrowhead), vestibule (black arrowhead), posterior semicircular canal (small arrow), and lateral semicircular canal (arrow) on the right (**a**) and left (**b**) sides. **c-d** Non-contrast HT2-FLAIR imaging: Abnormal high signal is depicted in the left vestibule (black arrowhead); this signal is higher than that of the CSF in the cerebellopontine angle cistern without sharply delineated borders (Grade 1). In contrast, the right inner ear shows low signal intensity, similar to the CSF in the cerebellopontine angle cistern, representing a normal signal (Grade 0). **e-f** Fusion images generated from MRC and non-contrast HT2-FLAIR image: The MRC image provides anatomical reference in grayscale, while the non-contrast HT2-FLAIR image is superimposed using a colormap to highlight regions of increased signal intensity. Abbreviations: 3D *three-dimensional*, FLAIR *fluid attenuated inversion recovery*, MRC *MR cisternography*, HT2-FLAIR *heavily T2‑weighted 3D FLAIR*, CSF *cerebrospinal fluid*.

**Fig. 2:**
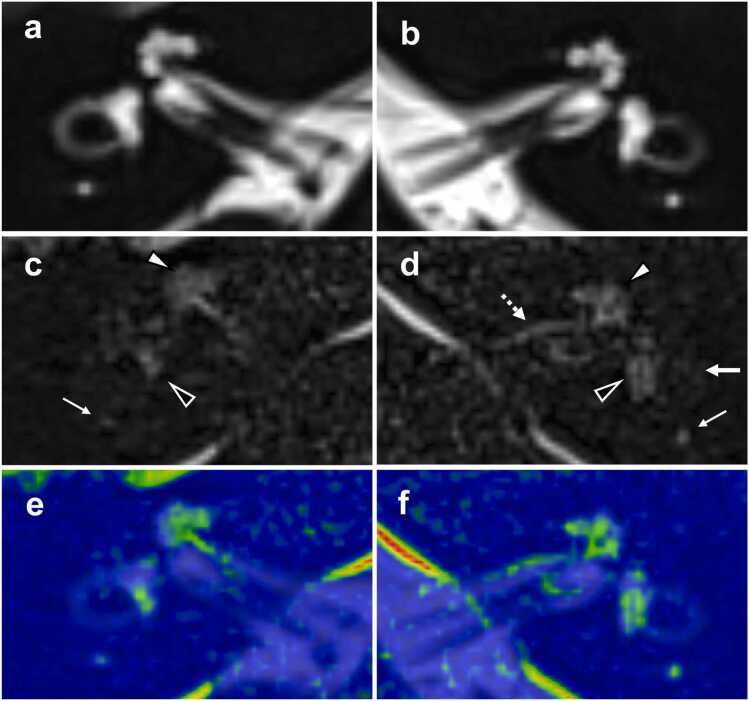
**68-year-old male with inner ear high signal on non-contrast 3D FLAIR imaging, associated with bilateral tinnitus. a-b** MRC: The inner ear lymph on the right (**a**) and left (**b**) sides demonstrates hyperintensity. **c-d** Non-contrast HT2-FLAIR imaging: Hyperintense signals are observed in the lymphatic fluid of both inner ears, including the cochlea (white arrowhead), vestibule (black arrowhead), posterior semicircular canal (small arrow), and lateral semicircular canal (arrow). Abnormal signals, all rated as grade 1, seem to be present within the perilymph. Notably, linear hyperintensity is evident within the left internal auditory canal (dotted arrow), indicative of dural/subdural lesions. **e-f** Fusion image generated from MRC and non-contrast HT2-FLAIR image. Abbreviations: 3D *three-dimensional*, FLAIR *fluid attenuated inversion recovery*, MRC *MR cisternography*, HT2-FLAIR *heavily T2‑weighted 3D FLAIR*.

**Fig. 3:**
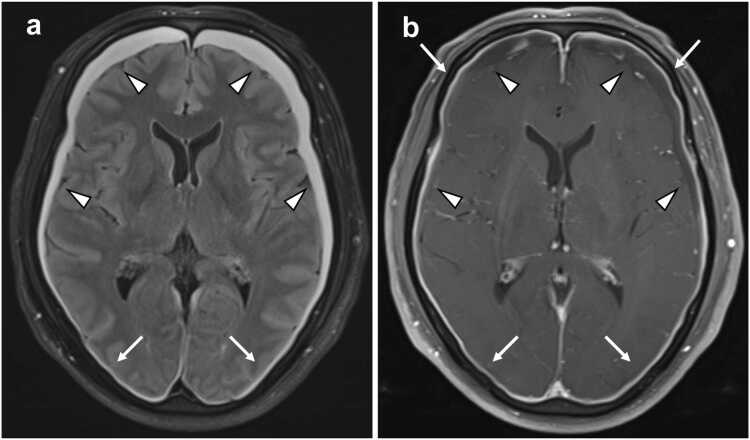
**68-year-old male with dural/subdural lesions (the same patient shown in**Fig. 2**). a** Non-contrast FLAIR imaging: Diffuse pachymeningeal hyperintensity (arrows) and subdural effusion/hematoma (arrowheads) are observed, although the two cannot be clearly distinguished. **b** Post-contrast T1-weighted imaging: The dura mater demonstrates diffuse enhancement and thickening, indicative of diffuse pachymeningeal enhancement (arrows). Subdural fluid without contrast enhancement is present, corresponding to subdural effusion/hematoma as identified on non-contrast FLAIR imaging (arrowheads). Abbreviations: FLAIR *fluid attenuated inversion recovery*.

**Fig. 4:**
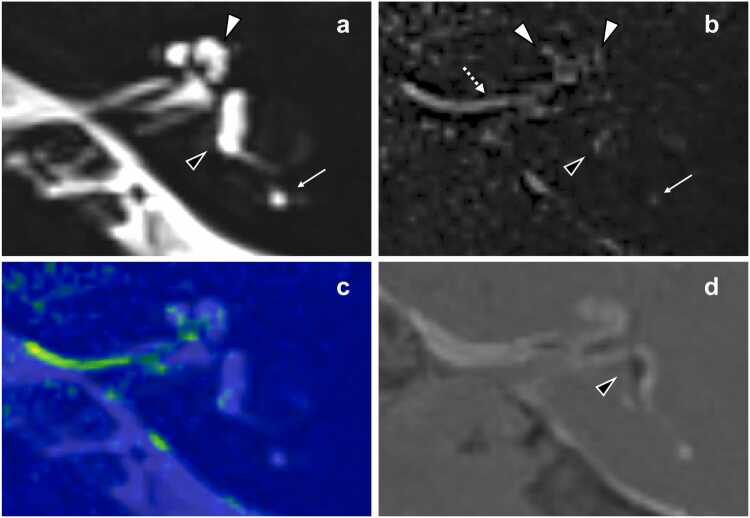
**50-year-old female with inner ear high signal and endolymphatic hydrops, associated with bilateral auditory symptoms (hearing loss, tinnitus, and ear fullness). a** MRC: Hyperintensity is observed within lymph fluid located in the left cochlea (white arrowhead), vestibule (black arrowhead) and posterior semicircular canal (arrow). **b** Non-contrast HT2-FLAIR imaging: Abnormal signals are depicted in the left cochlea (white arrowheads), vestibule (black arrowhead) and posterior semicircular canal (arrow). The vestibular high signal, located peripherally, seems to be within the perilymphatic space. Notably, linear hyperintensity is observed within the left internal auditory canal (dotted arrow), indicative of dural/subdural abnormality. **c** Fusion image generated from MRC and non-contrast HT2-FLAIR image**. d** 4-hour delayed contrast-enhanced HYDROPS: The left vestibule shows significant hydrops (black arrowhead), while the cochlea demonstrates no evidence of hydrops. Abbreviations: MRC *MR cisternography*, HT2-FLAIR *heavily T2‑weighted three-dimensional fluid attenuated inversion recovery*, HYDROPS *hybrid of reversed image of positive endolymph signal and native image of positive perilymph signal*.

All images were assessed in a blinded manner by two board-certified neuroradiologists (K.N. and I.O.). Independent reviews were performed, and any discrepancies in qualitative or semi-quantitative analyses were resolved by discussion to reach a consensus. High signal intensity on non‑contrast 3D FLAIR imaging was assessed in the cochlea, vestibule, and each semicircular canal (SCC) (anterior or superior, lateral or horizontal, posterior) bilaterally (Figs. 1 and 2). We visually evaluated the signal intensity of the lymph using the following 3-point-scale proposed by Osawa et al [12]. (Fig. 1): 0 = similar to that of CSF in the cerebellopontine angle cistern (normal signal), 1 = higher than that of CSF without sharply delineated borders, and 2 = markedly higher than that of CSF with sharply delineated borders. Grades 1 and 2 were defined as abnormal increased signal. Dural/subdural lesions were also assessed as diffuse pachymeningeal enhancement on post‑contrast T1-weighted imaging, and diffuse pachymeningeal hyperintensity and/or subdural effusion/hematoma on non-contrast FLAIR imaging (Fig. 3) [3]. Because the latter two findings identified on non-contrast FLAIR imaging were not able to be reliably differentiated using this modality alone, we categorized them collectively as diffuse dural/subdural hyperintensity. In cases where contrast-enhanced T1-weighted imaging was performed but non-contrast FLAIR imaging was not, subdural effusion/hematoma was assessed only using the contrast-enhanced T1-weighted images. Using non-contrast HT2-FLAIR imaging in addition to routine head MRI, we also assessed dural/subdural lesions in the internal auditory canal, indicative of hyperintensity (Figs. 2 and 4).

We semi-quantitatively assessed endolymphatic hydrops of the cochlea and vestibule according to the criteria proposed by Nakashima et al [16]. The grading system of cochlear endolymphatic hydrops was as follows: no = no bulging of the cochlear duct or no visualization of the cochlear duct, mild = the cochlear duct was bulging toward the scala vestibuli without exceeding the perilymphatic space of the scala vestibuli, significant = the cochlear duct exceeded the perilymphatic space of the scala vestibuli. Endolymphatic hydrops of the vestibule was graded as follows: no = less than 33% of the vestibule was occupied by the endolymph, mild = 33–50% of the vestibule was occupied by the endolymph, significant = more than 50% of the vestibule was occupied by the endolymph. We also assessed the presence or absence of brain sagging which was defined radiologically as tonsillar herniation 5 mm or greater below a straight line drawn between the basion and opisthion (McRae line) at the foramen magnum [17] on either sagittal cervical T1-weighted (TR 480–575 ms, TE 10–11 ms, slice thickness 3.0 mm) or T2-weighted (TR 1000–4000 ms, TE 83–97 ms, slice thickness 3.0–5.0 mm) images for the evaluation of spinal CSF leakage.

### 2.4 Statistical analysis

Statistical analyses were performed using Fisher’s exact test with a two-sided *p* value < 0.05 considered statistically significant. Associations between inner ear abnormal signal and dural/subdural lesions, cochlear signal and auditory symptoms (i.e. hearing loss, tinnitus, and ear fullness), and vestibular/SCC signal and vertigo were assessed. We also evaluated relationships between dural/subdural lesions in the internal auditory canal and audiovestibular symptoms, as well as between inner ear abnormal signal and corresponding endolymphatic hydrops. Associations excluding those between inner ear abnormal signals and dural/subdural lesions and those between vertigo and MRI findings were assessed not only at the patient level but also at the ear level. Analyses were performed using the statistical computing language R (Version 4.4.2; https://www.r-project.org/).

## 3 Results

Our study included 21 patients (42 ears) with CSF leaks, all of whom were diagnosed with SIH. The age was 37.5 ± 14.5 (mean ± standard deviation), and the sex was 11/10 (male/female). Clinical symptoms at presentation were as follows: hearing loss in 4 patients (19%; right in 1, bilateral in 3), tinnitus in 8 patients (38%; right in 1, left in 1, bilateral in 6), ear fullness in 7 patients (33%; right in 1, left in 1, bilateral in 5), vertigo in 8 patients (38%), and orthostatic headache in 20 patients (95%). Tinnitus was more frequently observed than hearing loss. Among all patients with tinnitus, 4 patients did not present with hearing loss. Inner ear high signal on non‑contrast 3D FLAIR imaging was identified in 13 patients (61%) (Fig. 2). The distribution of inner ear high signal (number of ears with signal) is shown in Table 1. Every high signal in the inner ear was classified as grade 1, which seemed to be mainly situated in the perilymphatic space. Dural/subdural lesions on head MRI were present in 11 patients (52%) (Fig. 3): diffuse pachymeningeal enhancement in 9, diffuse dural/subdural hyperintensity on FLAIR imaging in 8 (6 patients had both diffuse pachymeningeal enhancement and diffuse dural/subdural hyperintensity). In the subgroup of patients exhibiting diffuse pachymeningeal enhancement on contrast-enhanced T1-weighted images without non-contrast FLAIR acquisition, subdural fluid was not identified. Details of the patients are shown in Supplementary Table.

**Table 1:** Distribution of inner ear high signal on non‑contrast 3D FLAIR imaging (number of ears).

	Right	Left
Cochlea	10	11
Vestibule	9	10
Semicircular canal		
Anterior	6	5
Lateral	6	6
Posterior	7	6

Statistically significant association was observed between inner ear abnormal signal and dural/subdural lesions (*p* = 0.008) as shown in Table 2. Hearing loss and tinnitus showed a statistically significant association with cochlear abnormal signal in the ear-level analysis; however, no such association was observed with hearing loss in the patient-level analysis (Table 3). No association between ear fullness and cochlear abnormal signal was observed in either the ear-level or the patient-level analysis (Table 3). Furthermore, the relationship between vestibular/SCC abnormal signal and vertigo was insignificant (Table 3). Our analysis revealed no correlation between dural/subdural lesions in the internal auditory canal and audiovestibular symptoms at the ear and/or patient levels (Table 3). Abnormal signals in the cochlear and vestibule did not correlate with corresponding endolymphatic hydrops in either the ear-level or the patient-level analysis (Table 4). We considered it preferable to prioritize the patient-level interpretation as our primary analysis to ensure clinical robustness. While the ear-level results maintained granular, side-specific observations, they could be better interpreted as exploratory because two ears from the same patient may not be independent observations. Brain sagging was observed in only one case throughout the study.

**Table 2:** Relationship between inner ear abnormal signal and dural/subdural lesions.

		Dural/subdural lesions	
		Present	Absent
Inner ear abnormal signal (n = 21)	Present	10	3
	Absent	1	7
*p* value		**0.008**	
Odds ratio (95% CI)		19.2 (1.56–1142)	

CI *confidence interval*

Statistical analysis was performed using Fisher’s exact test. Bold font indicates a statistically significant result (*p* < 0.05).

**Table 3:** Audiovestibular symptoms in relation to abnormal inner ear signal and to dural/subdural lesions in IAC.

Unit of analysis	n	MRI finding	Symptom	MRI finding (+)/Symptom (+)	MRI finding (+)/Symptom (-)	MRI finding (-)/Symptom (+)	MRI finding (-)/Symptom (-)	*p* value	Odds ratio (95% CI)
Ear-level	42	Cochlear abnormal signal	Hearing loss	7	14	0	21	**0.009**	∞ (1.77–∞)
	42		Tinnitus	12	9	2	19	**0.003**	11.8 (2.02–131)
	42		Ear fullness	8	13	4	17	0.306	2.56 (0.35–44.1)
	42	Dural/subdural lesions in IAC	Hearing loss	3	11	4	24	0.669	1.61 (0.20–11.5)
	42		Tinnitus	6	8	8	20	0.490	1.85 (0.39–8.64)
	42		Ear fullness	4	10	8	20	1.000	1.00 (0.18–4.95)
Patient-level	21	Cochlear abnormal signal	Hearing loss	4	7	0	10	0.090	∞ (0.68–∞)
	21		Tinnitus	7	4	1	9	**0.024**	14.0 (1.15–782)
	21		Ear fullness	5	2	6	8	0.362	3.00 (0.35–44.1)
	21	Vestibular/semicircular canal abnormal signal	Vertigo	4	7	4	6	1.000	0.86 (0.11–7.01)
	21	Dural/subdural lesions in IAC	Hearing loss	5	6	2	8	0.362	3.14 (0.35–44.1)
	21		Tinnitus	6	5	2	8	0.183	4.43 (0.51–62.3)
	21		Ear fullness	4	7	3	7	1.000	1.32 (0.15–12.6)
	21		Vertigo	1	6	7	7	0.174	0.18 (0.00–2.14)

IAC *internal auditory canal*, CI *confidence interval*

Statistical analysis was performed using Fisher’s exact test. Bold values indicate statistically significant results (*p* < 0.05).

**Table 4:** Relationship between abnormal inner ear signal and endolymphatic hydrops.

Unit of analysis	n	Inner ear abnormal signal	Endolymphatic hydrops	Signal (+)/Hydrops (+)	Signal (+)/Hydrops (-)	Signal (-)/Hydrops (+)	Signal (-)/Hydrops (-)	*p* value	Odds ratio (95% CI)
Ear-level	26	Cochlea	Cochlea	3	9	7	7	0.248	0.35 (0.04–2.27)
	26	Vestibule	Vestibule	6	7	6	7	1.000	1.00 (0.17–2.11)
	52	Cochlea/vestibule	Cochlea/vestibule	9	16	13	14	0.413	0.61 (0.17–6.03)
Patient-level	13	Cochlea	Cochlea	2	4	4	3	0.592	0.41 (0.02–5.51)
	13	Vestibule	Vestibule	3	4	3	3	1.000	0.77 (0.05–10.7)
	26	Cochlea/vestibule	Cochlea/vestibule	5	8	7	6	0.695	0.55 (0.09–3.26)

CI *confidence interval*

Statistical analysis was performed using Fisher’s exact test.

## 4 Discussion

In our study, abnormal high signals were detected within the inner ear in patients with CSF leaks on non-contrast FLAIR imaging, appearing to be located in the perilymphatic space, and were statistically significantly associated with existence of dural/subdural lesions. Hearing loss and tinnitus statistically significantly correlated with cochlear FLAIR high signal in the ear-level analysis, while no statistically significant association was observed between the other audiovestibular symptoms and inner ear FLAIR hyperintensity. To the best of our knowledge, this is the first study to investigate non‑contrast FLAIR signal intensity changes within inner ear in patients with CSF leaks. In other ear disorders such as vestibular schwannoma, it has been shown that increased protein levels in the labyrinth cause FLAIR hyperintensity due to the T1 shortening of lymph fluid [12]. These signal changes are generally assumed to occur in the perilymph. There is a direct connection between the CSF and the perilymph through the cochlear aqueduct [18]; thus, decreased intracranial pressure in patients with CSF leaks can lead to a loss of perilymph volume through this connection [18]. We hypothesize that the resultant perilymph depletion increases the permeability of the blood labyrinthine barrier in order to create appropriate osmotic balance within inner ear fluid, resulting in elevated protein levels in the perilymph.

Another possible mechanism is venous congestion in the inner ear. CSF leakage can induce venous dilatation in the skull, which is explained as a compensatory mechanism by the Monro-Kellie doctrine. A Doppler ultrasound study evaluating straight sinus flow after lumbar puncture revealed a 47% reduction in mean velocity compared to pre-puncture levels. This finding suggested that decreased venous flow may contribute to cerebral venous thrombosis following lumbar puncture [19]. Given that the venous system of the inner ear communicates with intracranial veins [20], it is hypothesized that if the reduction in venous flow caused by CSF leaks extends to this system, venous congestion may occur within the inner ear. This could potentially increase the permeability of the blood labyrinth barrier. Under this hypothesis, intravascular proteins might leak into the lymphatic fluid, potentially resulting in high signal intensity on 3D FLAIR imaging. Since the blood perilymph barrier is typically more permeable than the blood endolymph barrier, such abnormal hyperintensity would be expected to appear predominantly in the perilymphatic space. Other possible mechanisms include immune- or inflammation-mediated responses, as well as reactions of the sympathetic nervous system. Although these mechanisms have been reported in other auditory disorders [21], [22], no reports have been found for CSF leaks.

There may be close pathological relationships between dural and subdural lesions associated with CSF leaks. The dural border cell layer, the innermost part of the dura mater, is believed to be the site of origin for both lesions [3]. Diffuse pachymeningeal enhancement has been hypothesized to result from the following mechanism: when CSF volume and pressure decrease, compensatory dilation of dural vessels—primarily veins—may occur in accordance with the Monro-Kellie doctrine. This potentially allows gadolinium-based contrast agent to accumulate within the dilated vessels located in the dural border cell layer. Because the dural vasculature lacks a blood brain barrier, the contrast agent could leak into the interstitium along the pressure gradient. It is also possible that FLAIR hyperintensity observed in the dura mater may result from extravasation of protein components from dilated dural vessels. In contrast, subdural effusion/hematoma might occur due to the extravasation of blood components from dilated dural vessels.

Subdural effusion is often associated with diffuse pachymeningeal enhancement, and is typically absent in areas lacking this pattern of contrast enhancement. This may suggest that subdural effusion occurs when compensatory dural venous dilation alone is insufficient to maintain intracranial volume [23]. Accordingly, subdural effusion could be interpreted as reflecting increased fluid accumulation within the dural border cell layer in response to potentially greater CSF leakage [24]. Our study has revealed that abnormal signals in the inner ear are associated with dural/subdural findings; thus, it is possible that decreased intracranial pressure is also related to changes in inner ear signals.

Our study demonstrated a correlation between cochlear FLAIR hyperintensity and hearing loss as well as tinnitus in the ear-level analysis, but no relationship was observed between inner ear hyperintensity and the other audiovestibular symptoms. Previous reports have indicated that tinnitus occurs more frequently than hearing loss in patients with CSF leaks [4], [5], and our findings were consistent with these observations. The cochlear perilymph is crucial for maintaining the electrical gradients and allowing the auditory hair cells to efficiently perform mechanoelectrical transduction of sound into neural signals [25]. Therefore, an increase in protein concentration in the perilymph may disrupt fluid equilibrium and potentially damage hair cells. In contrast to the ear-level results, hearing loss showed no significant association with cochlear abnormal signal at the patient level. This discrepancy between the-ear level and patient-level results can be explained by the difference in statistical independence between the two analytical units. Two ears from the same patient may not be independent observations. Therefore, ear-level Fisher’s exact tests may overestimate statistical significance because they treat bilateral ears as separate data points even though they share the same biological and clinical background. In contrast, the patient-level analysis collapses bilateral information into a single data point per patient, thereby accounting for the non-independence of the two ears and reducing the effective sample size and consequently decreases statistical power. This may be one of the reasons why the association between cochlear hyperintensity and hearing loss—detected in the more granular ear-level analysis—did not reach significance in the patient-level analysis. Given these statistical considerations, we considered it preferable to prioritize the patient-level interpretation as our primary analysis to ensure clinical robustness. While the ear-level results maintained granular, side-specific observations, they could be better interpreted as exploratory. However, the direction of association (as reflected by the odds ratios) was consistent across both levels of analysis, suggesting that the overall trend was similar even though statistical significance was not reached at the patient level. Further studies with larger cohorts and clustered analysis models are needed to validate these findings.

Endolymphatic hydrops has been proposed as a potential mechanism underlying audiovestibular symptoms in patients with CSF leaks; however, our study found no correlation between inner ear high signal and endolymphatic hydrops. If abnormal high signal in the inner ear were to reflect a compensatory increase in protein concentration to maintain osmotic pressure in response to decreased perilymphatic pressure, it is conceivable that this mechanism could be activated in some cases with abnormal hyperintensity, potentially leading to improvement of endolymphatic hydrops. Other hypothesized mechanisms to explain ear symptoms of CSF leaks include traction of the cochleovestibular nerve and dural thickening in the internal auditory canal. As our study demonstrated tonsillar herniation observed in only one case, traction of the cochleovestibular nerve is less likely to explain these symptoms. Dural thickening in the internal auditory canal has been reported in patients with SIH. Whether the dural thickening causes ear symptoms is still debated. Isildak et al. proposed that the irritation of the cochleovestibular nerve may be a cause of audiovestibular symptoms in patients with SIH [8]. In contrast, Hori et al. demonstrated no correlation between hearing disturbance and dural thickening in the internal auditory canal [26]. The present study showed no correlation between dural/subdural lesions in the internal auditory canal and audiovestibular symptoms, which is less likely to explain these symptoms. An additional mechanism, as previously discussed, is venous congestion of the inner ear. It is hypothesized that CSF leaks can induce intracranial venous dilation, potentially affecting the venous drainage of the inner ear and resulting in venous engorgement or stasis. This could theoretically lead to circulatory insufficiency and subsequent inner ear dysfunction [20].

The origin and underlying pathophysiology of tinnitus is still debated. Tinnitus can be divided into two major categories based on the hypothesized site of tinnitus generation: peripheral (i.e. cochlea and cochlear nerve) and central (i.e. auditory brain center) origins. While the peripheral origin of tinnitus was previously emphasized, the central origin is now widely recognized [27]. One prevailing theory for the central origin of tinnitus is that tinnitus arises from increased central excitability as compensation for the loss of peripheral input from the cochlea [28]. Patients with tinnitus frequently have hearing loss, and studies have identified a strong association between tinnitus parameters and auditory thresholds [29]. In contrast, tinnitus can occur in individuals with normal hearing, and some patients with tinnitus exhibit no detectable hearing loss using standard audiological tests [28]. Our study demonstrated that 50% (4/8) of the tinnitus patients did not present with hearing loss. If tinnitus in patients with CSF leaks originates from the peripheral auditory system [27], inner ear abnormal signal might play a role in its pathogenesis. Conversely, previous reports revealed that cochlear impairments and hyperactivity in the central auditory pathway in tinnitus patients with normal hearing, supporting the hypothesis that decreased neural activity from a damaged cochlea can elicit hyperactivity from decreased inhibition in the central nervous system [28]. Therefore, if inner ear abnormal signals observed in patients with CSF leaks are indeed associated with cochlear impairments even in the presence of normal hearing, these findings might support the hypothesis regarding a central contribution to tinnitus.

The vestibular/SCC high signal intensity on FLAIR images did not show a statistically significant association with vertigo. In the prior works concerning vestibular schwannoma, FLAIR signal alteration in the vestibular perilymph is not statistically significantly correlated with vertigo [12]. However, this is controversial, because vestibular/SCC abnormality appeared to correlate with vertigo in some case series [30]. Further research is needed to examine the relationship between vestibular/SCC signal abnormality and clinical manifestations.

HT2-FLAIR sequence, a variant of 3D FLAIR sequence that has gained widespread clinical use [31], was utilized in our study as the imaging sequence for detecting abnormal signals in the inner ear. Compared to conventional 3D FLAIR sequence, HT2-FLAIR sequence offers greater sensitivity to mild T1 shortening effects and is applicable not only in 4-hour delayed post-contrast MRI but also in non-contrast MRI [12]. It has also been applied to regions outside the inner ear, such as intracranial CSF spaces [32], [33] and spinal CSF leaks [1]. As the inner ear signal abnormalities observed in our study were subtle, HT2-FLAIR imaging may allow visualization of subtle changes within the inner ear that conventional 3D FLAIR imaging fails to detect.

The present study had several limitations. Firstly, this study is limited by its retrospective single‑center design, small sample size, and exclusions that reduced the analytic cohort. Second, since the absence of a control group limits our ability to establish the precise diagnostic specificity of these findings, the observed hyperintensities are better regarded as clinical associations within the context of CSF leaks at this stage. Nevertheless, in patients with unilateral vestibular schwannoma, it has been reported that the overall mean grade of inner ear signals on the unaffected side was nearly zero, significantly lower than the 1.45 observed on the affected side [12]. This finding supports the premise that the high signals identified in our cohort likely reflect a pathological departure from the normal physiological state. Third, because an ear-level analysis may not treat the two ears of the same patient as independent observations, there is a risk that statistical significance could be overestimated. Fourth, inner ear signal assessment was qualitative and based on visual grading without quantitative validation or interobserver agreement metrics. Given the subtle nature of inner ear FLAIR signal changes, observer bias cannot be excluded. Fifth, MRI sequence heterogeneity—particularly variability in the availability of non‑contrast FLAIR and post‑contrast T1‑weighted imaging—may have influenced the detection of dural/subdural lesions. Sixth, the audiovestibular symptoms were not characterized in detail and were heterogeneous. In addition, we did not include standardized audiometric or vestibular testing results because of the restricted number of cases available for analysis, thereby limiting the strength of imaging–clinical correlations. Seventh, as CSF pressure was not assessed in all patients in our study, a direct association between CSF pressure and abnormal inner ear signals cannot be definitively determined. Nevertheless, CSF pressure may differ between the initial phase of CSF leakage and the compensatory phase mediated by the Monro-Kellie mechanism. Notably, CSF pressure in patients with SIH is known to vary widely, ranging from low to normal or even elevated [34]. Thus, the absence of such direct correlation does not exclude the potential impact of CSF pressure reduction. Eighth, we evaluated tonsillar herniation by measuring caudal displacement from a defined anatomical reference. However, brain sagging may have occurred even in cases not meeting the criteria for tonsillar herniation, although its evaluation is challenging without pre-onset imaging. Finally, the mechanistic interpretations we propose are best regarded as hypotheses that may help guide further investigation, rather than as definitive explanations. Overall, our results may be best regarded as exploratory and hypothesis-generating, and future prospective studies with larger cohorts, standardized testing, quantitative imaging approaches, and appropriate control groups would be valuable.

## 5 Conclusions

Non‑contrast 3D FLAIR hyperintensity in lymph fluid of cochlea/vestibular structures is observed in patients with CSF leaks and correlates with dural/subdural imaging findings. Primary patient-level analysis reveals a significant correlation between tinnitus and cochlear high signal; however, the association with hearing loss is not observed at this level. While exploratory ear-level analysis suggests that both symptoms may correlate with cochlear findings, these results—particularly concerning hearing loss—could be interpreted with caution and require further validation in larger studies. Overall, these observations may support the clinical utility of non-contrast 3D FLAIR imaging as a practical imaging tool of inner ear involvement in CSF leak patients.

## CRediT authorship contribution statement

**Iichiro Osawa:** Writing – original draft, Methodology, Formal analysis, Data curation, Conceptualization. **Takashi Mitsufuji:** Writing – review & editing, Supervision, Data curation. **Keita Nagawa:** Writing – review & editing, Supervision, Formal analysis, Data curation. **Yuya Yamamoto:** Writing – review & editing, Supervision. **Hirokazu Shimizu:** Writing – review & editing, Supervision. **Genko Oyama:** Writing – review & editing, Supervision. **Shinji Kakemoto:** Writing – review & editing, Supervision. **Kaiji Inoue:** Writing – review & editing, Supervision. **Nobuo Araki:** Writing – review & editing, Supervision. **Eito Kozawa:** Writing – review & editing, Supervision.

## Informed consent

This retrospective study was conducted at a single institution and approved by the Institutional Review Board of our institution. We obtained written informed consent for the procedures and opt-out consent for the use of retrospective clinical data from all patients/ from parents or legal guardian of patient less than 18 years.

## Ethical approval

All procedures performed in studies involving human participants were in accordance with the ethical standards of the institutional and/or national research committee and with the 1964 Helsinki declaration and its later amendments or comparable ethical standards.

## Funding

This research was supported by JSPS KAKENHI under Grant Number JP22K15807, Health and Labour Sciences Research Grants under Grant Number 23GC1002 and 26GC1701, and Grant-in-Aid from Saitama Medical University Internal Research under Grant Number 21-B-1–05.

## Declaration of Competing Interest

The authors declare that they have no conflict of interest.

## Footnotes

Appendix A Supplementary data associated with this article can be found in the online version at doi:10.1016/j.ejro.2026.100758.
