# Supplementary material for: Inner ear high signal on non‑contrast 3D FLAIR imaging in patients with cerebrospinal fluid leaks: Association with dural/subdural changes and with audiovestibular symptoms
Source: Eur J Radiol Open. 2026 May 4;16:100758. doi: 10.1016/j.ejro.2026.100758 (PMC13157017; doi:10.1016/j.ejro.2026.100758)
Supplement: Supplementary file 1 — Supplementary material [file mmc1.docx]

**Supplemental File:**

**Supplementary table.** Summary of 21 patients with cerebrospinal fluid leaks

| No. | Age (years) | Sex | Etiology | CSF pressure (cmH_2_O) | Symptoms | |  |  | |  | |  | | Dural/subdural lesions |  | Dural/subdural lesions in IAC | Inner ear high signal on non-contrast 3D FLAIR imaging |  |  |  |  | Endolymphatic hydrops |  |  |  | Tonsillar herniation |
| --- | --- | --- | --- | --- | --- | --- | --- | --- | --- | --- | --- | --- | --- | --- | --- | --- | --- | --- | --- | --- | --- | --- | --- | --- | --- | --- |
|  |  |  |  |  | Hearing loss | Tinnitus | | | Ear fullness | | Vertigo | | Orthostatic headache | DPE | DDSH |  | Cochlea | Vestibule | Anterior SCC | Lateral SCC | Posterior SCC | Cochlea |  | Vestibule |  |  |
|  |  |  |  |  |  |  | | |  | |  | |  |  |  |  |  |  |  |  |  | R | L | R | L |  |
| 1 | 29 | F | SIH | 0 | 0 | 1 (L) | | | 1 (L) | | 0 | | 1 | 1 | 1 | 1 (B) | 1 (B) | 0 | 0 | 0 | 0 | NA | NA | NA | NA | 0 |
| 2 | 28 | M | SIH | NA | 1 (B) | 1 (B) | | | 1 (B) | | 0 | | 1 | 1 | 0 | 0 | 1 (B) | 1 (B) | 0 | 0 | 0 | 1 (M) | 0 | 1 (S) | 1 (M) | 0 |
| 3 | 40 | F | SIH | NA | 1 (B) | 1 (B) | | | 1 (B) | | 0 | | 1 | NA | 1 | 1 (B) | 1 (B) | 1 (B) | 1 (B) | 1 (B) | 1 (B) | 0 | 0 | 1 (M) | 1 (M) | 1 |
| 4 | 50 | F | SIH | NA | 1 (R) | 1 (R) | | | 1 (R) | | 0 | | 1 | 1 | 1 | 1 (B) | 1 (B) | 1 (B) | 1 (B) | 1 (B) | 1 (B) | 0 | 0 | 1 (M) | 1 (S) | 0 |
| 5 | 68 | M | SIH | 5 ≤ | 0 | 1 (B) | | | 0 | | 1 | | 0 | 1 | 1 | 1 (B) | 1 (B) | 1 (B) | 1 (B) | 1 (B) | 1 (B) | NA | NA | NA | NA | 0 |
| 6 | 48 | F | SIH | NA | 0 | 0 | | | 0 | | 0 | | 1 | NA | 1 | 0 | 1 (L) | 1 (L) | 0 | 1 (L) | 0 | NA | NA | NA | NA | 0 |
| 7 | 32 | F | SIH | NA | 1 (R) | 1 (B) | | | 1 (B) | | 1 | | 1 | 0 | NA | 0 | 1 (B) | 1 (B) | 0 | 0 | 1 (B) | 1 (M) | 1 (M) | 0 | 0 | 0 |
| 8 | 25 | F | SIH | NA | 0 | 0 | | | 0 | | 1 | | 1 | 0 | NA | 0 | 0 | 1 (B) | 0 | 0 | 0 | 1 (S) | 1 (S) | 1 (S) | 1 (S) | 0 |
| 9 | 40 | M | SIH | 12 | 0 | 0 | | | 0 | | 0 | | 1 | 1 | NA | 1 (B) | 1 (B) | 1 (B) | 0 | 0 | 0 | 0 | 0 | 0 | 0 | 0 |
| 10 | 32 | M | SIH | NA | 0 | 0 | | | 0 | | 0 | | 1 | 0 | NA | 0 | 0 | 1 (L) | 0 | 0 | 0 | 0 | 0 | 0 | 0 | 0 |
| 11 | 43 | M | SIH | NA | 0 | 0 | | | 0 | | 0 | | 1 | 1 | 1 | 1 (B) | 1 (B) | 1 (R) | 1 (R) | 1 (R) | 1 (R) | NA | NA | NA | NA | 0 |
| 12 | 69 | M | SIH | NA | 0 | 1 (B) | | | 0 | | 1 | | 1 | 1 | 1 | 0 | 1 (B) | 1 (B) | 1 (B) | 1 (B) | 1 (B) | 0 | 0 | 0 | 0 | 0 |
| 13 | 30 | M | SIH | NA | 0 | 0 | | | 0 | | 0 | | 1 | 1 | NA | 1 (B) | 1 (B) | 1 (B) | 1 (B) | 1 (B) | 1 (B) | NA | NA | NA | NA | 0 |
| 14 | 30 | F | SIH | NA | 0 | 1 (B) | | | 1 (B) | | 1 | | 1 | 0 | 0 | 0 | 0 | 0 | 0 | 0 | 0 | 1 (S) | 1 (S) | 1 (S) | 1 (S) | 0 |
| 15 | 16 | M | SIH | NA | 0 | 0 | | | 0 | | 1 | | 1 | 0 | NA | 0 | 0 | 0 | 0 | 0 | 0 | NA | NA | NA | NA | 0 |
| 16 | 37 | F | SIH | NA | 0 | 0 | | | 1 (B) | | 0 | | 1 | 0 | NA | 0 | 0 | 0 | 0 | 0 | 0 | 0 | 0 | 0 | 0 | 0 |
| 17 | 60 | F | SIH | 9.2 | 0 | 0 | | | 0 | | 1 | | 1 | 0 | NA | 0 | 0 | 0 | 0 | 0 | 0 | 1 (S) | 1 (S) | 1 (M) | 1 (M) | 0 |
| 18 | 31 | M | SIH | NA | 0 | 0 | | | 0 | | 0 | | 1 | 1 | NA | 0 | 0 | 0 | 0 | 0 | 0 | 0 | 0 | 0 | 0 | 0 |
| 19 | 19 | M | SIH | NA | 0 | 0 | | | 0 | | 1 | | 1 | NA | 0 | 0 | 0 | 0 | 0 | 0 | 0 | 0 | 1 (M) | 0 | 0 | 0 |
| 20 | 19 | F | SIH | NA | 0 | 0 | | | 0 | | 0 | | 1 | 0 | NA | 0 | 0 | 0 | 0 | 0 | 0 | NA | NA | NA | NA | 0 |
| 21 | 42 | M | SIH | NA | 0 | 0 | | | 0 | | 0 | | 1 | 0 | NA | 0 | 0 | 0 | 0 | 0 | 0 | NA | NA | NA | NA | 0 |

CSF *cerebrospinal fluid*, IAC *internal auditory canal*, 3D FLAIR *three-dimensional fluid attenuated inversion recovery*, DPE *diffuse pachymeningeal enhancement*, DDSH *diffuse dural/subdural hyperintensity*, SCC *semicircular canal*, R *right*, L *left*, SIH s*pontaneous intracranial hypotension*, 1 *positive*, 0 *negative*, B *bilateral*, NA *not available*, M *mild*, S *significant*
